# Deep-Learning-Based Antenna Alignment Prediction for Mobile Indoor Communication

**DOI:** 10.3390/s23073375

**Published:** 2023-03-23

**Authors:** Árpád László Makara, Botond Tamás Csathó, András Rácz, Tamás Borsos, László Csurgai-Horváth, Bálint Péter Horváth

**Affiliations:** 1 Department of Broadband Infocommunications and Electromagnetic Theory, Faculty of Electrical Engineering and Informatics, Budapest University of Technology and Economics, Műegyetem rkp. 3., H-1111 Budapest, Hungary; 2 Ericsson Research, H-1117 Budapest, Hungary

**Keywords:** deep learning, mmWave, antenna beam alignment, ray tracing, mobile indoor communication

## Abstract

A significant innovation for future indoor wireless networks is the use of the mmWave frequency band. However, an important challenge comes from the restricted propagation conditions in this band, which necessitates the use of beamforming and associated beam management procedures, including, for instance, beam tracking or beam prediction. A possible solution to the beam management problem is to use artificial-intelligence-based procedures to learn the hidden spatial propagation patterns of the channel and to use this knowledge to predict the best beam directions. In this paper, we present a deep-neural-network-based method that has memory that can be used to predict the best reception directions for moving users. The best direction is the highest expected signal level at the next moment. The resulting method allows for a user-side antenna management system. The result was evaluated using three different metrics, thus detailing not only its predictive ability, but also its usability.

## 1. Introduction

Complementing sub-6 GHz frequency bands [1] with the millimetre Wave (mmWave) spectrum is already happening in 5G, and it is expected to continue in future 6G systems as well, mainly due to the scarcity of spectrum resources in the lower bands. In order to take full advantage of the mmWave bands, there are a few challenges that need to be solved, one of them being the difficult propagation conditions, which require orienting signal transmission in an adequately selected beam direction in order to reach the intended receiver. This means beamforming and the selection of the right beam direction both at the Base Station (BS) and at the User Equipment (UE) side.

Beam scanning is the process during which the BS and UE select the beam directions that result in the best channel conditions, which need to be continuously updated as the UE is moving or the surrounding environment is changing. In the worst case, when there are *N* and *M* possible beam directions at the BS and at the UE, respectively, we need N×M beam measurements to select the best direction. One simple way to speed up this process is hierarchical beam scanning, as also introduced in 3GPP 5G procedures [2]. However, the best beam directions may change suddenly due to environment-specific reflections, blockages, etc. In such cases, the time spent with a hierarchical or full scan search may result in a connection drop and disruption in communication. Therefore, many researchers have turned toward Artificial-Intelligence (AI)-based solutions that try to predict changes in the best beam directions ahead of time or predict the best beam directions from a few probing measurements in some designated beam directions [3]. The 3GPP has also initiated a new study item recently for Rel.18 with the aim of investigating the reduction of beam management overhead by the use of AI [4,5].

The importance of beam management may increase in future systems, e.g., with dense deployments of BSs, by installing a significantly higher number than in today’s system. More UE mobility or with dense, complex environments with no or a limited Line-of-Sight (LOS) and quickly changing propagation paths both indoors and outdoors requires also more advanced beam alignment. To summarise, the primary problem that needs to be solved is to ensure that the mobile user is always connected to the base station. Furthermore, a key objective is to ensure that the devices can reach the highest possible data speed during the connection. In this type of interconnection, a higher received signal level results in a better signal-to-noise ratio; therefore, maximising this amount satisfies both objectives. Of course, it is also possible to increase the SNR by choosing the right cells with the right deployment of BSs [6], but for better testability, we always used one BS in the present study. The hardest part of the problem is that the UE can move arbitrarily and Non-Line-of-Sight (NLoS) connections may occur, which needs to be handled by the system.

The design of the latest mobile network systems requires that connections can utilise the highest amount of available bandwidth and servicing as many devices as possible. In this context, one of the possible and necessary solution is the use of antennas and antenna systems operating in multiple frequency bands [7]. This allows data to be always transmitted in the frequency band that is best suited to the circumstances. Moreover, in a given frequency band, it is possible to implement beamforming using antenna systems. However, in the present research, only a specific fixed frequency band was investigated, without addressing the limitations of antennas.

Indoor localisation is a long-researched topic, which shows some similarities with the topic of the current research, but there are also significant differences. The most-significant difference is that our solution does not care where the UE is located or in which room it can be found, only where the antenna beam should be directed. However, the procedures and considerations highlighted in [8] may be also advantageous, where various Neural Network (NN) solutions were applied to Bluetooth Low Energy signals for angle-of-arrival-based position determination. Their best performing solution is based on a convolutional NN, which requires intensive data preparation. The most-relevant result of this research was that the best-performing model of the mesh network has 20,000 parameters, i.e., it can learn this amount of variables.

In this work, we not only focused on designing some new algorithm for a particular use case, but rather, we made an attempt to establish an understanding of the theoretical limitations of performing such beam predictions with AI. We wanted to see what the endpoints of the scale are in terms of prediction performance as a function of the number of probing measurements. We investigated how much the training process impacts the performance and how the training samples should be collected to obtain an accurate model. A third and essential aspect investigated in the paper is how much the AI models trained in one propagation environment can be generalised to other environments, as this would be an essential aspect for the practical deployment of such solutions. In order to obtain results that are as close to reality as possible, we relied on a combination of Ray Tracing (RT) simulations of some selected real environments and combined these simulations with real measurements performed in the same environment with our own purpose-built measurement equipment operating at 38.8 GHz [9].

It was presented in the literature that neural networks can learn the main features of an indoor propagation environment, which are presumably based on a similar solution to pattern-based tasks. For example, the authors of [10] applied a Deep Neural Network (DNN) to model indoor propagation accurately. On this basis, we investigated NN-based solutions that may be suitable for solving the current problem. In the present theoretical procedure (which used a DNN), the moving UE performs the antenna beam alignment task based on the measured signal levels of the BS.

The paper structure is as follows: In Section 2, we detail the problem area to be resolved and the applied methods and tools. After this, in Section 3, the most-important results are presented, and finally, in Section 4, a summary of the results is given.

## 2. Problem Formulation

Indoor communication links can be improved through several strategies. This is because about 80% of wireless communication traffic happens indoors [11]. Among the possible solutions, the authors of [12] used a DNN-based approach similar to the solution that we present. Beam and blockage prediction was implemented in the procedure. The procedure was based on the 5G New Radio models. The solution in terms of DL is essentially a mapping process that combines online and offline learning. The DNN structure is memoryless, consisting only of fully connected and dropout layers. A relatively small amount of data, 11,200, was used for the training. The attached results showed that, in the tested cases, the individual nets performed exceptionally well. The most-crucial difference between this procedure and ours is that it includes the specific location information for the user, but our solution does not utilise this kind of information.

The goal of our solution is to estimate the best beam direction for communication (where the received signal level will be the highest at the given point that can be available) in the next time slot based on the beam probing measurements of the UE in the previous time slots. By probing measurements, we mean that the instrument measures in fixed, defined directions. For this purpose, we used a DNN, which accepts only the measured signal levels as the input in some selected probing directions. In other words, the UE does not know either where it is or where the BS is. Furthermore, this information will not be needed during its operation. We assumed that the beamforming is carried out at the UE, since the measured signal levels are available there, while the BS has a fixed beam position. Nevertheless, by reversing the problem, the beamforming task could be easily transferred to the BS, by assuming the presence of UE–BS service communications. Presumably, such a transformation would not affect the presented results significantly; therefore, within this study, this case is not discussed.

To train the NN, simulated data were applied, derived from our RT simulations and validated with measurement results [13]. As the method is based on time series, not only the locally measured signal levels are carrying information, but also the order in which they are sampled. These samples are called “movements”, and their choice and structure greatly affect the capabilities of the algorithm [14]. This modification is essential because it helps in better approximating how users are moving. Therefore, one of the advantage of this method is that it considers the expected movements of users in the estimation.

In the rest of this section, we describe the individual units of the procedure and their most-important properties.

### 2.1. Measurement Based Indoor Ray Tracing Simulation

The datasets for the NN were generated with an open-source, flexible RT called Q-D Realization [15]. Besides, we also made a measurement campaign in a standard office room with dimensions of 6.5 × 8.8 m to study the propagation conditions of an indoor channel [9]. In the simulation, this physical size was the starting point for the room dimensions, which were divided into about 20k simulated points with equidistant 5 cm spacing. The results can also be used to validate the utilised RT tool. In Q-D Realization, isotropic antennas are assumed; therefore, post-processing was developed in Matlab to encounter the radiation patterns. For the sake of brevity, here, we recall the key considerations; the interested reader is referred to [13].

First, we approximated the radiation pattern with a Gaussian beam. The parameters of the beam were tuned to obtain the best fit between the measurement and simulation. Second, the ray tracer evaluates possible propagation paths with the corresponding delays. These components were summarised phase coherently to encounter interference. Third, even though the measurements were carried out at 38.8 GHz, we utilised a material library of Q-D Realization created for 60 GHz, due to the similar dielectric properties of common building materials (brick) [16].

**Figure 1:**
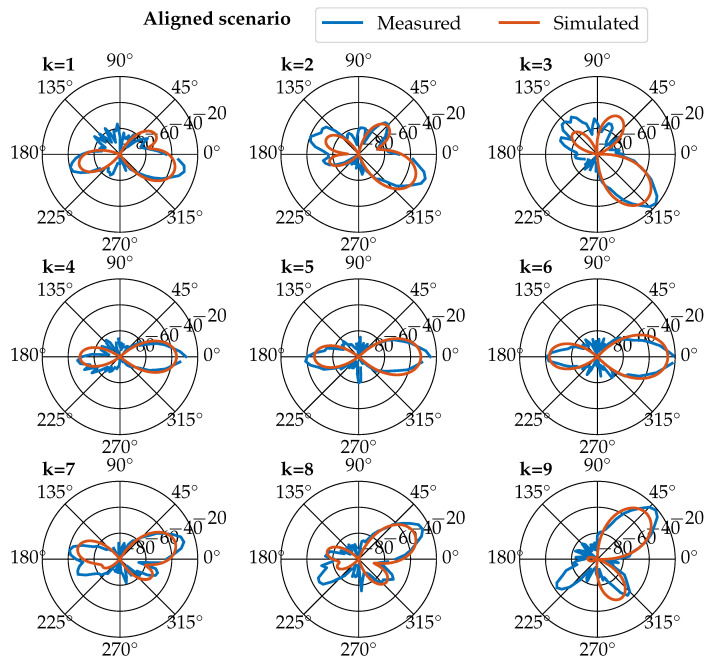
Power patterns. Sub-figures are positioned relative to each other to indicate RX positions in the room [13].

A part of the result is shown in Figure 1, which shows a comparison between real measurements at reference points and simulation results.

For the measurements, one transmitter and one receiver antenna were used. The position of the transmitter (the BS) was fixed, and the receiver (the UE) was moved in the room. We measured the received power pattern in a horizontal plane. The tested scenario was the aligned scenario [13], where the transmitter was always directed toward the receiver’s position, which is actually a simplified theoretical model of how the BS has already performed the necessary beamforming.

### 2.2. The Prediction Structure and the Problem to Solve

**Figure 2:**
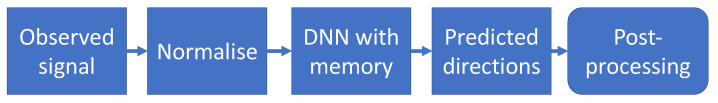
The used prediction structure.

The operation of the applied DNN is based on the signal level measured from a few selected probing directions. The user device estimates the direction from which the highest signal level is expected in the next time slot. A schematic diagram of the procedure is shown in Figure 2. It is known from previous research relating to indoor propagation that, based on these measurements, at a given frequency, it is possible to fit a model that well indicates the signal-receiving conditions [17]. If we know the direction of the next highest available signal level or if we can obtain this information with some post-processing, we can perform beam alignment with an algorithm based on these data.

The input signal, a four-element vector, is called: x(Δt), where Δt is the sampling time (which is used to indicate that the sampled values are assumed to be constant over a chosen time window). Each element of the vector is a received signal level in dBm. The i-th element of this vector always represents a fixed direction (angle), which in this case are $0^{\circ }$ (first element of the vector etc.), $90^{\circ }$, $180^{\circ }$, and $270^{\circ }$. These directions are fixed with respect to the room; in fact, they are bound to the BS, as shown in the polar diagrams in Figure 1.

The interpretation of the output is a bit more complex, starting by dividing the plane into *n* equal parts. These are the individual output directions, centred on the point where the controlled antenna (UE) is directed. Each constructed domain is adjacent to two neighbours, henceforth denoted by $r^{i:k}$, where *i* is the initial angle of the range and *k* is the final angle at the given point. The sum of the ranges covers the entire space, without overlaps and, for simplicity, referred to by sequential number starting from the domain that includes the $0^{\circ }$ range. In our research, we most often used a division into 18 ranges, each 20 degrees wide.

It is necessary to assign a value to each range, in dBm, of course, in order to use it for supervised machine learning. Of the available options, we used the nominal value of the range, i.e., the reception value associated with the centre element, which is the signal level measured at the centre of the UE antenna (if it were pointing towards it). At a given point, the values of the ranges form a vector, in this case an 18-element vector, denoted R. The elements of this vector are the (output) directions. At a given point, the best choice is the element with the highest value, which is called then the best direction (which is a number or, in other words, a category), marked with *D*:

(1) $$D_{p}=\mathrm{number}\left(R_{p},\max\left(R_{p}\right)\right),$$

where *p* indicates the given point, number is a function that gives the serial number of the element of the vector to which the second attribute of the function belongs. For clarity, the first direction is adjacent to the second direction and the last, in this case, the eighteenth, the others being, by definition, the one less and one more.

To summarise, the method assumes an algorithm (denoted by F) that solves the following problem at each point:

(2) $$D_{p}\left(\Delta t+1\right)=F\left(x_{p}\left(\Delta t\right)\right),$$

where *p* indicates at that point. Categorising the problem to be solved, the procedure is a real-time prediction that uses a time series as the input and a transform of the time series as the output. The procedure is considered universal if *F* works with acceptable accuracy for all possible *p*.

### 2.3. The Structure of the Used Deep Neural Network

**Figure 3:**
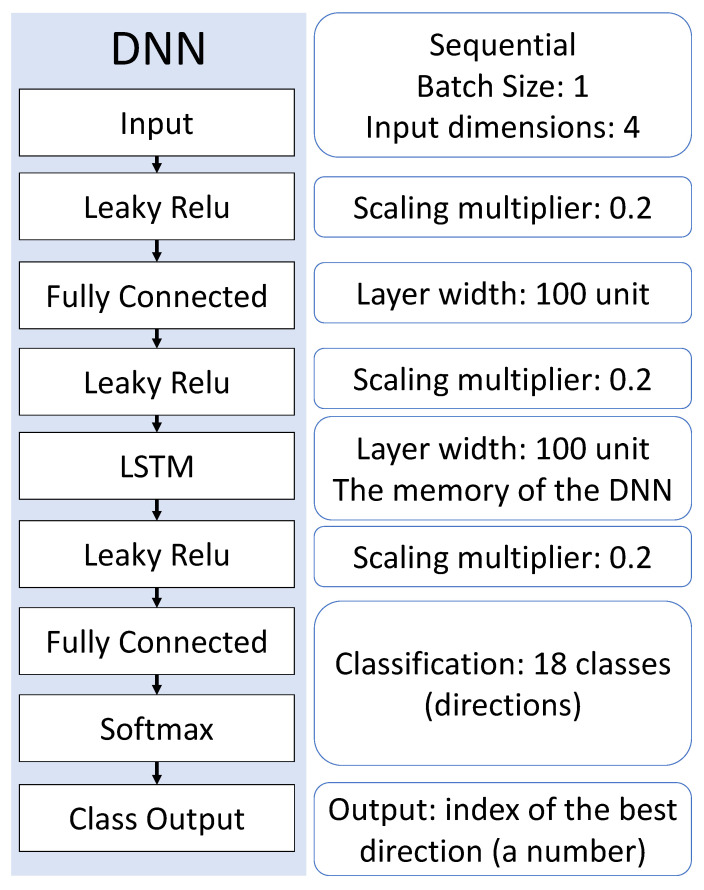
The used DNN structure.

In the design, we aimed to apply the smallest, narrowest structure for the DNN that can solve the presented problem. The applied DNN layout is shown in Figure 3, and the parameters were applied. The main element of a recurrent neural network is the use of the Long Short-Term Memory (LSTM) layer, which is able to find correlations between successive input elements in the time series by means of its internal states (also, it has memory) [18,19]. Although the LSTM layer may be able to perform scaling and feature selection tasks [19], due to the larger amount of parameters that can be taught, a fully connected layer was used before. Classification was performed by the fully connected layer after the LSTM layer and the softmax function [20]. The softmax function assigns a quasi-likelihood to each possible direction (so the sum of each value is always 1), while the subsequent class output layer makes a hard decision to choose the maximum of this vector [18,21]. The entire structure has approximately 82.5 k learnable parameters, giving a total of 100 internal states connected in a chain. To achieve a valid training, we applied a time series of about 1 million elements. For training, the Adam optimiser [22] was used with the cross-entropy cost function [23]. During the normalisation of the input data, the following algorithm is applied in every time step:

(3) $$x_{\mathrm{norm}}\left(\Delta t\right)=\frac{x_{\mathrm{measured}}\left(\Delta t\right)-\mu_{\Delta t}}{\sigma_{\Delta t}},$$

where $x_{norm}$ is the normalised value, μ is the mean, and σ is the standard deviation of $x_{measured}$ [24]. The purpose of this amendment is to help the DNN learn from relative relations.

According to our investigations, one of the best-performing structures as shown in Figure 3 was improved compared to the previous versions. For each layer, the Leaky Rectified linear unit layer (Leaky Relu) activation function was placed with a scaling factor of 0.2 [25]:

(4) $$L\left(x_{i}\right)=\left\{\begin{array}{cc} x_{i}, & \mathrm{if}\;x_{i}\geq 0,\\ scale\;\cdot \;x_{i}, & \mathrm{if}\;x_{i}<0, \end{array}\right.$$

where $x_{i}$ is the current input vector’s i-th element. The first application of Leaky Relu is easy to explain: For the problem applied, the input signal levels in dBm are negative. Thus, the normalisation by the mean (which is also negative) transforms the values above the mean into the positive range and leaves the activation function unchanged, while the values below the mean are shifted. This suggests that the neural network performs better when the above-average values are from a wider dynamic range. In our experience, the use of additional Leaky Relu layers also improves the accuracy of the network, but it is more complex to know exactly what is being transformed.

### 2.4. Main Parameters of the Procedure

A supervised machine learning framework learns on the training data [24]. For this reason, it matters how we generate the training dataset. Based on the above, the following parameters are relevant [13,14,26]:

- User velocity/sampling frequencies;
- Number of output classes;
- Used received signal directions;
- Used accuracy metric;
- User movement strategy during the training;
- Aligned/unaligned scenario;
- Room structure.

Other factors may also have an influence on the prediction capabilities, but according to our experience, the above-mentioned parameters are the most-relevant.

#### 2.4.1. User Velocity and the Sampling Frequencies

Depending on how fast each of the input data arrive, the accuracy of the trained neural network varies [13]. This can occur due to one of two reasons: the speed at which the user is moving or the sampling frequency of the user position. In the movement simulation for training data generation, the two cases may be considered as identical. Apart from the initial tests, we always worked with a constant data rate/measurement rate for a better overview of the problem area.

#### 2.4.2. Sampling Distance

This process depends on the speed at a large scale. The distance between the consecutive sampling points significantly influences the accuracy of the prediction [13]. The sampling distance can vary because of the variation of the UE’s speed or the probing frequency. Apart from the initial tests, we always worked with a constant sampling distance. The smaller the sampling distance is, the more accurate the estimation is [13]. However, there were also exciting results when testing and training were performed with different sampling distances. In all cases, the accuracy degraded, especially when using a smaller sampling distance with the NN trained on a larger sampling distance. In conclusion, a future problem to be solved is to find a universal solution that works independently of sampling distance.

#### 2.4.3. Number of Output Classes and Used Input Directions

The accuracy and complexity of the system is determined by how many outputs, how many inputs, and how many probing directions there are. Our experience showed that the more inputs we used, the better the results would be. In the case where 8 input signals were used instead of 4, approximately a 2–4% increase in accuracy was obtained. In other words, the use of additional inputs typically does not bring as much gain as much extra computation is required.

As far as the outputs are concerned, estimating fewer categories is basically more accessible. However, on the basis of the results so far, 18 direction categories can be estimated with reasonable accuracy, each covering an area roughly equal to the beamwidth of the applied antenna [17]. The use of the fewest possible directions is also of particular importance since, in practice, it is likely that the same antenna will be used for each measurement. The more measurements there are, the more resource-intensive they are, and the more likely the environment is to change in the process. In summary, the beamwidth of the antennas should be proportional to the number of categories used and the size of the ranges they cover. Naturally, if an omnidirectional antenna is used, beamforming is not necessary.

In the current solution, for the sake of simplicity, we used directions fixed relative to the room for both the output and input. However, this is also a parameter to be tested and investigated to see how our method may perform in a moving coordinate system.

#### 2.4.4. Received Signal Directions and Accuracy Metric

The direction of the received signal and the accuracy metric are two distinct, but interrelated parameters. The reason that only a finite number of classes or directions were selected is that the direction space is discretised. It is necessary to assign a value to each direction to implement supervised machine learning [24]. We obtained different results depending on whether it is a nominal value from the range, an average value, or a weighted value within a range. However, we are confident that their impact will not be significant.

Depending on how we interpret the accuracy, we can use the assumption to reduce the errors mentioned earlier. Three interpretations were tested: simple accuracy, TOP3 accuracy, and adjacent accuracy [13,26]. To show the individual metrics, in the Δt-th time step, we denote the predicted direction by the serial number $P_{p}\left(\Delta t\right)$, where *p* in the subscript denotes the given point, since in the case of a static transmitting antenna, only the position dependence is of primary importance.

Simple Accuracy (SA) is the ratio of correct predictions to total predictions; at one point, it is the following:

(5) $${\mathrm{SA}}_{p}\left(\Delta t\right)=\left\{\begin{array}{cc} 1, & \mathrm{if}\;P_{p}\left(\Delta t\right)=D_{p},\\ 0, & \mathrm{if}\;\mathrm{otherwise}. \end{array}\right.$$

This is, in fact, the accuracy of the quickest method to interpret, where the predicted value is taken as the correct one without any posterior search.

One possible method to avoid errors is to compare the three most-probable values of the softmax layer in the TOP3 accuracy (TOP3) case with the correct result [20]; at one point, it is the following:

(6) $${\mathrm{TOP}3}_{p}\left(\Delta t\right)=\left\{\begin{array}{cc} 1, & \mathrm{if}\;P_{p}\left(\Delta t\right)=D_{p},\\ 1, & \mathrm{if}\;\mathrm{number}\left(R_{p},\max2\left(R_{p}\right)\right),\\ 1, & \mathrm{if}\;\mathrm{number}\left(R_{p},\max3\left(R_{p}\right)\right),\\ 0, & \mathrm{if}\;\mathrm{otherwise}, \end{array}\right.$$

where max2,max3 are functions that always give the second- or third-largest value of the vector; this is, in fact, a simulation of how the most-probable values of the UE are checked by the system in the post-processing. Of course, this can be interpreted for any number of classes and will always give as good or better results than simple accuracy [13,14,26]. However, this approach requires an additional logical part in terms of beamforming.

In the case of Adjacent Accuracy (AA), the neighbours of the estimated most-probable values are also examined to deal with the particular issue where the discretisation would have cut a peak value in two by chance. One point can be interpreted as follows, where *n* is the last serial number of categories and *k* is the starting serial number:

(7) $${\mathrm{AA}}_{p}\left(\Delta t\right)=\left\{\begin{array}{cc} \mathrm{if}\;P_{p}\left(\Delta t\right)=n:\; & \left\{\begin{array}{cc} 1, & \mathrm{if}\;P_{p}\left(\Delta t\right)=n,\\ 1, & \mathrm{if}\;P_{p}\left(\Delta t\right)=n-1,\\ 1, & \mathrm{if}\;P_{p}\left(\Delta t\right)=k,\\ 0, & \mathrm{if}\;\mathrm{otherwise}, \end{array}\right.\\ \mathrm{if}\;P_{p}\left(\Delta t\right)=k:\; & \left\{\begin{array}{cc} 1, & \mathrm{if}\;P_{p}\left(\Delta t\right)=k,\\ 1, & \mathrm{if}\;P_{p}\left(\Delta t\right)=n,\\ 1, & \mathrm{if}\;P_{p}\left(\Delta t\right)=k+1,\\ 0, & \mathrm{if}\;\mathrm{otherwise}, \end{array}\right.\\ \mathrm{otherwise}:\;\; & \left\{\begin{array}{cc} 1, & \mathrm{if}\;P_{p}\left(\Delta t\right)=D_{p},\\ 1, & \mathrm{if}\;P_{p}\left(\Delta t\right)=D_{p}-1,\\ 1, & \mathrm{if}\;P_{p}\left(\Delta t\right)=D_{p}+1,\\ 0, & \mathrm{if}\;\mathrm{otherwise}, \end{array}\right. \end{array}\right.$$

which takes advantage of the fact that the first and last numbered categories are adjacent. This is the model where the estimated direction only suggests which neighbours need to be scanned. Of course, this is always better or equal to simple accuracy, but no correlation was found with TOP3 accuracy [26]. The advantage of this method is that it eliminates some of the possible errors due to discretisation, and if the individual ranges are thinner than the UE antenna beam, this method may be the most-practical.

It makes sense to apply any distance metric to an entire route by summing the metrics per point and dividing by the total number of estimates.

#### 2.4.5. Movement Strategy

**Figure 4:**
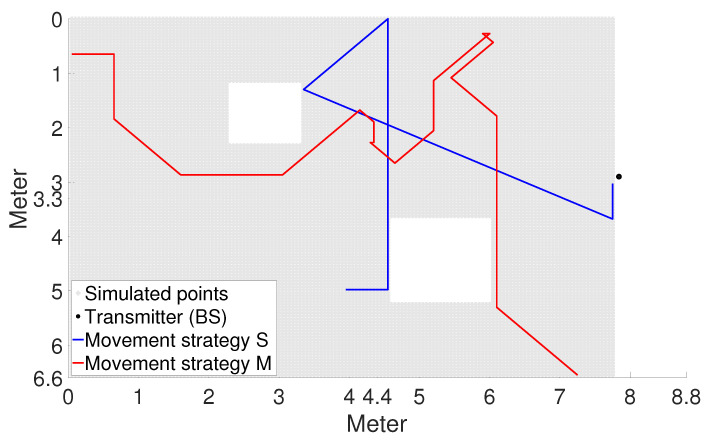
Comparison of movement strategies. Both users start from a random location. Their speed is the same, 5 cm/step. In the case of the Markov method, a behavioural profile that promotes frequent straight-line movement was suitable [14].

One of the most-complex and -complicated issues is the movement pattern and strategy during the training process (Figure 4). Depending on how we interpret this, the NN exhibits different prediction capabilities [14]. Our primary expectation was that the system should handle all the movements that may occur during the operation. The classic movement strategy (S strategy) is a simple straight-line progression with a flexible collision at any angle.

The solution to the problem is the combination of several different movement patterns during training and learning them all. At the same time, this brings up new questions on how to make the concatenations so that they do not cause hidden errors in the system. The best approach so far seems to be making all movements using some state-based Markov chain method (M strategy), where the geometry of the room and the user’s behavioural profile (which is called the Movement Behaviour Profile (MBP)) determine the probabilities of each transition [14]. The Markov chain method provides a good human-like movement simulation. The main steps of the procedure are as follows [14]:

- At each point, determine the distance to the nearest impenetrable object.
- A normalised vector is formed from the distances associated with the possible directions of motion (the sum of the vector is 1).
- According to the number of possible step directions, we form normalised vectors, which are the corresponding members of the previous vector and the MBP vector [14].
- From the generated vectors, we form one-to-one Markov chain transition matrices.
- Generating data using matrices: a Markov chain with a matrix of moving user arrivals is used to generate the next arrival point.

To simulate an arbitrary number of different motion trajectories in the same geometry room, the MBP is required. The latter is a vector that gives the probability that a given UE will move in each direction (or possibly stop). In the current phase of the research, the same MBP was used throughout to examine the other factors.

#### 2.4.6. Aligned and Unaligned Scenario

Both the aligned and unaligned scenarios are exceptional cases of how the BS antenna might be positioned [13]. Although the UE side currently performs the direction estimation, this can be symmetrically reversed in the current situation.

The unaligned scenario means that one of the antennas does not support beamforming and is fixed at one point. However, this case raises the question of what kind of antennas are used and where they are pointed by default. Of course, this case always performs worse [13].

During this research work, an aligned scenario was considered. The transmitting antenna always points straight at the receiver, also when there is an impenetrable barrier in the path, such as, e.g., a pillar. This method is easy to implement and straightforward and provides the best result.

#### 2.4.7. The Structure of the Rooms

Last, but not least, one important question is the possibility of generalisation. Experience so far has shown that the NN best performs in the same room where it was trained. If we test it in a slightly modified room, two questions arise: Why should it work, and what do we mean by a slightly modified room?

For each room and movement, assign a discrete probability distribution as follows [26]. From the interpreted best directions per point, construct a matrix, where each element of the matrix corresponds perfectly to its relative position with respect to the other points, for a motion, a line vector (time-interpreted time series), denoted by D. Let DN denote the total number of elements of the matrix (taking one dimension as one in the case of a vector), then:

(8) $$P_{D}\left(M\right)=\left\{\begin{array}{c} p_{1}=\frac{1}{DN}\;\cdot \;\sum_{x,y,D_{x,y}=1}D_{x,y},\\ p_{2}=\frac{1}{DN}\;\cdot \;\sum_{x,y,D_{x,y}=2}D_{x,y},\\ \vdots \\ p_{N}=\frac{1}{DN}\;\cdot \;\sum_{x,y,D_{x,y}=N}D_{x,y}, \end{array}\right.$$

where *N* is the number of the directions (in this case, 18) and M is the associated probability variable. Even the question of how to measure the difference between the rooms is not self-evident. Thus far, the total variation distance [27] between distributions interpreted in the best directions seems to be the best choice. However, parameters that have not yet been fully explored also influence this issue [26].

The most-complex topic is how a general solution can be found. We aimed for the DNN to learn local, relative environmental information, the features of which are independent of a specific room, depending only on environmental reflections and the distance from the transmitter. One of the main goals of our current research was how to achieve a general solution and how to qualify the performance of the DNN.

## 3. Results

To qualify the neural-network-based solution and determine the effect of individual parameters, we should investigate the right and wrong prediction results. From our former research, we know that there is a slight correlation between the AA and the probability distribution assigned to the movement (which is the distribution of $D_{p}$s) [26].

From the point of view of use, the most-important thing is how the pre-trained DNN performs in a general environment. In order to investigate this, we tested the trained DNN in three different rooms: A1, where the original measurements were performed and the DNN was trained with ray tracing data, A2, where we changed the position of the transmitter, and A3, where a third, additional impenetrable pillar object was added.

Our minimum expectation before carrying out the tests was that in rooms other than the training place, the accuracy decreased only along the distances. In addition, we expected that similar types of errors would occur in similar types of places. Of course, our ultimate goal was to provide a universal solution that works well in any indoor environment.

### The Impact of Changes in the Environment

The first question is how our trained network (hereafter, M net) performs. As a reference, we considered the trivial solution where the system knows the best direction at a given moment. When we suppose that the estimated direction for the next instant of time is identical to the previous one, after performing hundreds of thousands of test runs (based on the S and M strategies), the average accuracy was close to 70%, so this value was used as a reference accuracy.

We have seen so far that the total variation distance [27] is the most-descriptive metric for the distribution distance $D_{p}$s of two rooms, which is based on the following [26]:

- Distance between Rooms A1 and A2: 0.63.
- Distance between Rooms A1 and A3: 0.23.
- Distance between Rooms A2 and A3: 0.66.

Of particular interest is how these distances are influenced by an additional impenetrable object in the room. Because the DNN was trained with the movement sequences in A1, we expected that it would perform better in the room with less distance from the original. Along with the metrics used, this means the following:

- Distance between Room A1 and M net training data: 0.23.
- Distance between Room A2 and M net training data: 0.61.
- Distance between Room A3 and M net training data: 0.27.

Based on this, we can expect that M net will perform slightly poorer in the A3 room and much worse in the A2 room.

**Figure 5:**
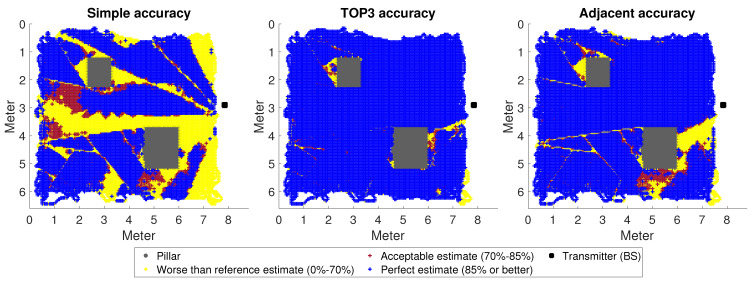
A movement of 5,000,000 steps in Room A1; the accuracy interpreted according to the metrics is presented. The areas marked in white were not visited during the test.

**Figure 6:**
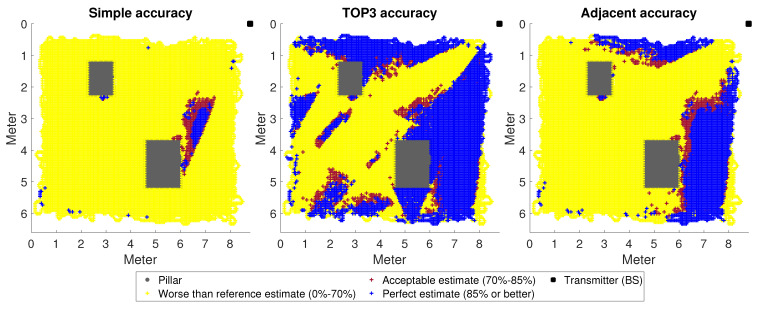
A movement of 5,000,000 steps in Room A2; the accuracy interpreted according to the metrics is presented. The areas marked in white were not visited during the test.

**Figure 7:**
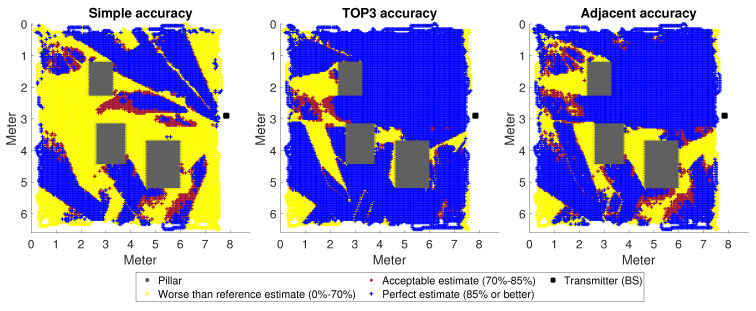
A movement of 5,000,000 steps in Room A3; the accuracy interpreted according to the metrics is presented. The areas marked in white were not visited during the test.

To test the results numerically, we used five million sample movements, an order of magnitude larger than our previous tests. For the most-accurate comparison, a random state of M net was saved. Each test was started from this random state. The individual predictions were summed and plotted as points, as shown in Figure 5 for Room A1, Figure 6 for Room A2, and finally, Figure 7 for Room A3. The results were divided into three categories: worse than the benchmark (which was previously 70%), medium (70–85%), and excellent (above 85%). It is understood that the really good scores are the scores in the last category, and the ultimate goal would be to have all predictions fall into this category.

The distribution of categories was far from even. The occurrence of the best (which was ultimately our goal) and the worst categories was much more frequent. All this suggested that the DNN was either always sure of making the right decision in a given situation or almost always made the wrong one. There were few areas where these two processes were roughly balanced.

The first thing to notice is that, as the distance increased, the SA decreased, as expected. The TOP3 metric performed best in all cases, giving quite acceptable results for A1 and A3. Although the current initial problem was simplified and the neural network learning was quite straightforward, the procedure itself suggested that the system could be good for decision support. What we mean by this is that a neural network trained with several movements from a more diversified space can presumably predict the most-likely directions with a good enough ratio.

Of particular note is the AA metric. By accepting the neighbouring classes of the estimated direction as correct, we improved the results by orders of magnitude. This could mean two things: on the one hand, that it could theoretically work to use narrower ranges than the antennas we want to use permanently to train the DNN, thus solving this situation; on the other hand, this may in fact mean that there is a better way of adding value to each category.

Last, but not least, the case of Room A2 is interesting (Figure 6), which stands out from A1 and A3. According to the distance metric presented, the neural network was worse clustered here. At the same time, the system also missed points in the prediction that can be considered trivial at the theoretical level, where the reflection from the columns (a kind of more free space) can be considered negligible. This was particularly striking in the case of the SA metric, where the misestimation rate was over 80%, which is beyond the distance of about 0.6. Although further investigation is needed, it is possible that the metric used is the most-accurate for the same BS position, so this is part of a more accurate description of the difference between rooms.

Beyond the numerical results, other key observations can be made. Some of these findings can be attributed to the evaluation of the results, while others were based on our experience in the learning process:

- Procedures without location information work for this kind of problem.
- Simultaneous UE and BS antenna beamforming can easily become chaotic. Presumably, each mobile device will have at most one antenna/antenna system at a given frequency. Thus, using the presented method, multiple measurements at different positions are required. During this time, the BS should not change its beam position. The synchronisation of this procedure is questionable.
- UE side calculations are resource intensive, simply due to the size of the DNN. It is questionable what the most-efficient implementation might be.
- Applying an online learning process can be also considered, using a net that has already been taught.
- Columns and other impenetrable objects significantly impair accuracy in NLoS cases, especially in unknown rooms. Among other things, this is due to destructive interference at the signal level. However, there are solutions to reduce interference, so the use of such procedures on the UE side is necessary (there is much 5G-related research of this kind, such as [28]).

## 4. Conclusions and Future Plans

Our presented technique is a candidate to solve the beam alignment problem when using a millimetre wavelength frequency band for indoor mobile communications. In doing so, the least-possible type of input data is used, such as the received signal level, which the UE can simply measure. The measurement of location data or other extended information is problematical at the UE side. The method estimates the best reception direction in the next time step with reasonable accuracy. The built environment has a strong influence on the results, and we proved that, by the metric calculations, the differences between various indoor environments can be expressed. In order to improve the precision of the NN-based beam alignment procedure and ensure the operation in a significantly different environment, a more complex retraining can be considered, by taking into account some additional parameters that have been identified in this research work.

A further research direction is to define a more detailed user scenario. The location of the beamforming (user equipment or base station), the applied discretisation method, the input dataset, the sampling rate, etc., have a significant impact on the results and the learning ability of the deep neural network. Thus, this is definitely the next necessary step toward a universal solution.

Based on related publications, one possible development of the method could be the use of online learning. This would compare the real measurement results with the output vector of the softmax layer and train the mesh with the calculated error. However, considering the computational capacity and the energy used, we would probably obtain a better result if all adaptive processes were performed on the BS side; therefore, the UE can be as simple as possible.

## Data Availability

Not applicable.

## Abbreviations

The following abbreviations are used in this manuscript:

AA	Adjacent Accuracy
AI	Artificial Intelligence
BS	Base Station
DNN	Deep Neural Network
Leaky Relu	Leaky Rectified linear unit layer
LoS	Line-of-Sight
LSTM	Long Short-Term Memory
MBP	Movement Behaviour Profile
ML	Machine Learning
mmWave	millimetre Wave
NLoS	Non-Line-of-Sight
NN	Neural Network
RT	Ray Tracing
SA	Simple Accuracy
TOP3A	TOP3 Accuracy
UE	User Equipment
